# Complete mitochondrial genome of *Hygrobates turcicus* Pešić, Esen & Dabert, 2017 (Acari, Hydrachnidia, Hygrobatoidea)

**DOI:** 10.1038/s41598-022-26188-w

**Published:** 2022-12-21

**Authors:** Andrzej Zawal, Lidia Skuza, Grzegorz Michoński, Aleksandra Bańkowska, Izabela Szućko-Kociuba, Romain Gastineau

**Affiliations:** 1 Institute of Marine and Environmental Sciences, Center of Molecular Biology and Biotechnology, University of Szczecin, Waska 13, 71–415 Szczecin, Poland; 2 Department of Plant Protection, Faculty of Agriculture, University of Tehran, PO Box 4111, Karaj, 31587/11167 Iran; 3 Institute of Biology, Center of Molecular Biology and Biotechnology, University of Szczecin, Waska 13, 71–415 Szczecin, Poland

**Keywords:** Computational biology and bioinformatics, Genetics, Molecular biology, Zoology

## Abstract

The aim of the study was sequencing of the mitogenome of *Hygrobates turcicus* Pešić, Esen & Dabert, 2017 to expand knowledge of the polymorphism and cryptic or pseudocryptic diversity within Hydrachnidia. The samples originated from Bulgaria, Vidima River near Debnewo, 42°56′41.4′′N, 24°48′44.6′′E, depth 0.4 m, stones on the bottom, water flow 0.71 m/s, temperature 10 °C, pH 8.53, oxygen 110%, conductivity 279 µS/cm, hardness 121 CaO mg/l; 11 males, 27 females, 2 deutonymphs 12.x.2019 leg. Zawal, Michoński & Bańkowska; one male and one female dissected and slides mounted. The study was carried out using the following methods: DNA extraction, sequencing, assembly and annotation, comparison with other populations of *H. turcicus*, and multigene phylogeny. As a result of the study, it was determined that the mitogenome is 15,006 bp long and encodes for 13 proteins, 2 rRNAs, and 22 tRNAs. The genome is colinear with those of *H. longiporus* and *H. taniguchii*, the difference in size originating from a non-coding region located between protein-coding genes *ND4L* and *ND3*. Five genes have alternative start-codon, and four display premature termination. The multigene phylogeny obtained using all mitochondrial protein-coding genes unambiguously associates *H. turcicus* with the cluster formed by *H. longiporus* and *H. taniguchii*.

## Introduction

Water mites (Hydrachnidia) are very diverse and species rich group of macroinvertebrates. They occupy almost all freshwater environments. An updated version of Limnofauna Europaea (www.watermite.org) shows the improvement of the knowledge on European water mite biodiversity. From the 1062 species listed in 1978 year, 28% have been synonymized or excluded because of their uncertain status (species incertae), while at the same time over 200 species were added^1^. There is still a clear gap in alpha-taxonomy and knowledge upon phenotypic polymorphism and cryptic diversity within Hydrachnidia. Recent publications^2–10^ indicate the presence of many unrecognized species, especially in the southern part of Europe, which can be distinguished by molecular methods.

The publication of the three parts of an identification key^11–13^ initiated a new trend in researchers on European water mites, facilitating or enabling ecological and biological research. The release of these keys was preceded by numerous revisions of individual genera^14–16^ based on morphological data. However, the development of molecular barcoding^17^ quickly suggested that alpha-taxonomy is still weakly recognised and there are many species-complex containing cryptic or pseudo-cryptic species. An important work on integrative taxonomy and phylogeny of water mites was published by Dabert et al.^18^. Their analyses, based on nuclear ribosomal genes such as 18S, 28S and fragments of the mitochondrial gene of the cytochrome c oxidase subunit 1 (*cox1*), provided evidence about the relationships within the group. Recent taxonomical studies^2–8^ indicated a lack of knowledge about phenotypic polymorphism and cryptic or pseudo-cryptic diversity within Hydrachnidia. Therefore, more genetic data are needed. One of the ways to achieve this is to sequence complete mitochondrial genomes. There is an important, rapidly growing literature dedicated to the sequencing of complete mitogenomes, but despite this, up to now only 12 mitogenomes of Hydrachnidia have been made available and published^19–23^.

To participate in this effort, we have undertaken the sequencing of the complete mitogenome of *Hygrobates turcicus* Pešić, Esen & Dabert, 2017, a species belonging to genera extensively studied by DNA-barcoding^2–6,9,10^. We compared the characteristics of this mitogenome with those of *Hygrobates longiporus* Thor, 1898 and *Hygrobates taniguchii* Imamura, 1954, and used the data to perform a multigene phylogeny.

## Results

### Mitogenome of *H. turcicus*

**Figure 1:**
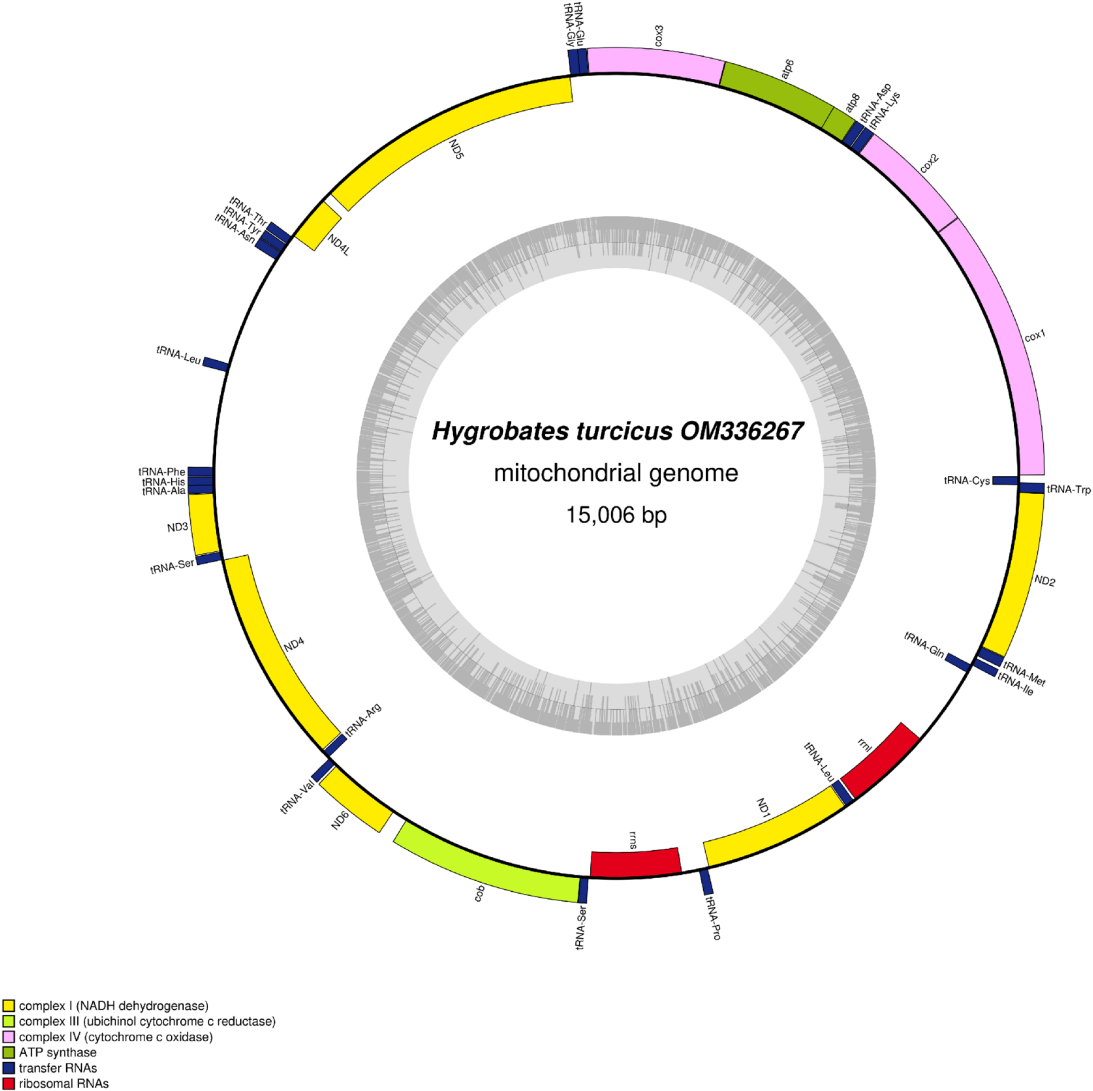
Map of the mitochondrial genome of *Hygrobates turcicus*. Genes belonging to different functional groups are color coded differently and the GC, AT content of the genome are plotted on the inner circle as dark and light gray, respectively.

The mitogenome of *H. turcicus* (GenBank accession number OM336267) is 15,006 bp long (Fig. 1) (Table 1). It contains 13 conserved protein-coding genes, two rRNAs, and 22 tRNAs. It is colinear with the mitogenomes of *H. longiporus* and *H. taniguchii*, but its size is ca. 1300 bp longer (Table 1). The extra-length originates mostly from a non-coding region, which is located between protein-coding genes *ND3* and *ND4L*. A comparison of the length, start and stop codon of the protein-coding genes of *Hygrobates* spp. is presented in Table 2. Two genes of *H. turcicus* present a premature termination, namely *cox2* and *cox3*. In the case of *cox3*, this is common to the three species of *Hygrobates* spp. The distribution of the start codons is 7 ATG, 3 ATT, 2 ATA and 1 TTG. This TTG start codon was found in the *ND5* gene, which discriminates *H. turcicus* from *H. longiporus* and *H. taniguchii*. A canonical start codon could not be identified in this case.

**Table 1:** List of the mitogenomes used during this study with their accession number and sizes.

Species	Accession number	Size of the mitogenome (in base pairs) (bp)
*Hygrobates turcicus*	OM336267	15,006
*Hygrobates longiporus*	LC552026	13,721
*Hygrobates taniguchii*	LC552027	13,770
*Leptotrombidium akamushi*	AB194045	13,698
*Leptotrombidium deliense*	AB194044	13,731
*Leptotrombidium pallidum*	AB180098	16,779
*Mideopsis roztoczensis*	MT671492	13,989
*Riccardoella reaumuri*	LC601993	15,148
*Riccardoella tokyoensis*	LC601992	15,078
*Sperchon plumifer*	MG701313	14,646
*Unionicola foili*	EU856396	14,738
*Unionicola parkeri*	HQ386015	14,734
*Walchia hayashii*	AB300500	14,857

**Table 2:** Comparison of the mitochondrial protein-coding genes of the three species of *Hygrobates*.

	*Hygrobates turcicus*			*Hygrobates longiporus*			*Hygrobates taniguchii*		
	Size	Start	Stop	Size	Start	Stop	Size	Start	Stop
*atp6*	663	ATG	TAA	663	ATG	TAA	663	ATG	TAA
*atp8*	147	ATT	TAA	147	ATA	TAA	147	ATG	TAA
*cob*	1098	ATG	TAA	1098	ATG	TAA	1068	ATT	TAA
*cox1*	1542	ATG	TAA	1545	ATG	TAA	1542	ATG	TAA
*cox2*	665	ATG	TA(A)	666	ATG	TAA	663	ATG	TAG
*cox3*	781	ATG	T(AA)	781	ATG	T(AA)	778	ATA	T(AA)
*ND1*	894	ATT	TAG	903	ATA	TAG	894	ATT	TAG
*ND2*	948	ATA	TAA	951	ATA	TAA	951	ATA	TAA
*ND3*	348	ATA	TAA	345	ATA	TAA	345	ATC*	TAA
*ND4*	1293	ATG	TAA	1275	ATG	TAA	1299	ATG	TAA
*ND4L*	276	ATG	TAA	288	ATA	TAA	298	ATA	T(AA)
*ND5*	1620	TTG	TAA	1654	CTT*	T(AA)	1654	CTT*	T(AA)
*ND6*	438	ATT	TAG	447	ATA	TAA	447	ATT	TAA

The table indicate the size of the genes (stop codon included) and the type of start and stop codons. The (A) and (AA) indicate an early termination with the stop codon being completed by the addition of 3' A residues to the mRNA. The * indicates that authors described the start codon as not being found (replaced here by the first codon of the gene).

### Comparison with other populations of *H. turcicus*

The percentages of identities between the available *cox1* genes of *H. turcicus* are listed in Table 3. These percentages range between 99.52 and 99.84%, with the highest percentage (99.84%) being found 10 times out of 16.

**Table 3:** Percentages of identity as calculated by Clustal Omega between the *cox1* gene obtained during this study and the same gene as available on GenBank.

GenBank accession number	Percentage of identity with XXX
KY609986	99.52
KY609980	99.52
KY609972	99.52
KY609976	99.84
KY609977	99.68
KY609971	99.68
KY609984	99.84
KY609983	99.84
KY609982	99.84
KY609979	99.84
KY609978	99.84
KY609975	99.84
KY609974	99.84
KY609973	99.84
KY609981	99.68
MN520308	99.84

### Multigene phylogeny

**Figure 2:**
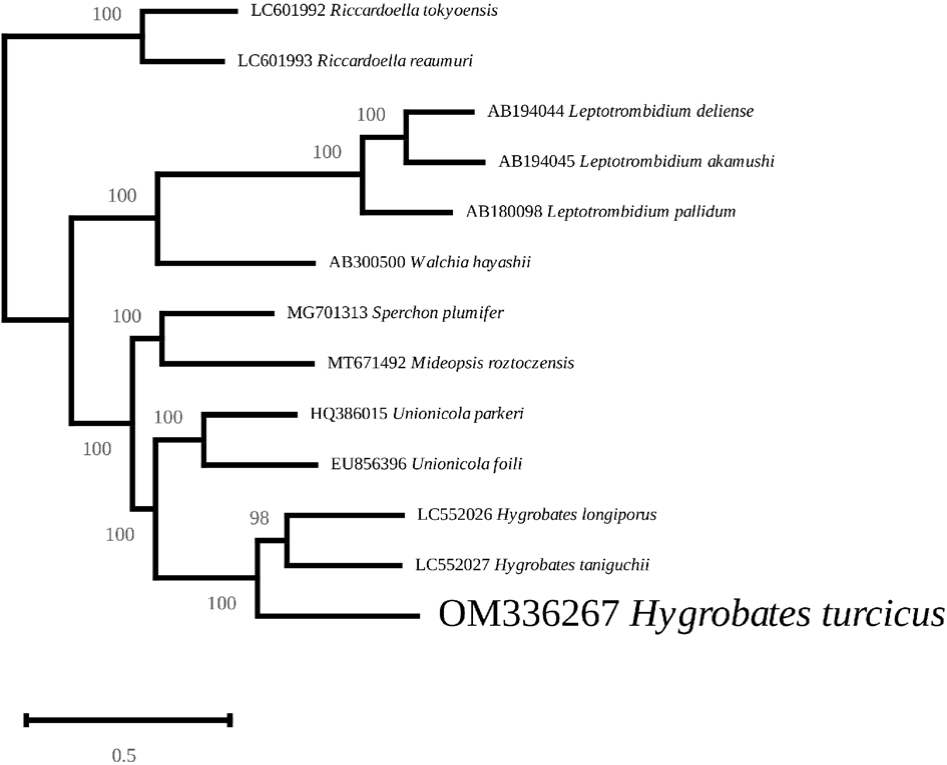
Cladogram illustrating the phylogenetic relationships for *Hygrobates turcicus* based on complete mitochondrial genome sequences. Mitochondrial genome rearrangement events are mapped on the branches of the best scoring maximum likelihood tree generated with RAxML-NG. Each node has 100% bootstrap support value.

The phylogenetic tree inferred from concatenated mitochondrial protein-coding genes displays high bootstrap values, ranging from 98 to 100%. The snail parasites *Riccardoella* spp. appear as an outgroup. Phylogenetic analyses clustered the analysed Parasitengona species into 6 maximally supported clades, three of them (1. *Sperchon plumeifer* and *Mideopsis roztoczensis*; 2. *Unionicola parkeri* and *U. foili*; 3. *H. longiporus*, *H. taniguchii* and *H. turcicus*) corresponding to the water mites. *H. turcicus was* recovered with high statistical support (98%) as a sister branch to *H*. *longiporus* and *H. taniguchii* (Fig. 2).

## Discussion

*H. turcicus* is a species distinguished recently on the basis of DNA-barcoding from the group of *H. fluviatilis-*complex species. It was described from Turkey and next mentioned from Bulgaria^3,5^. It is closely related with *H. ulii* Pesic, Saboori, Zawal & Dabert, 2019, and together with *H. balcanicus* Pesic, 2020, it is a separate clade in relation to the other species of *H. fluviatilis*-complex^5,6^. It is worth noting that based on *cox1* comparisons (Table 3), the specimen sequenced in the current study didn’t exhibit a higher conservation with the other specimen from Bulgaria (GenBank: MN520308), more precisely from the Strymon river, than with 9 others specimens originating from Turkey. This might prove to be a limitation for accurate biogeographical studies that need to be considered when using single gene barcoding.

Molecular barcoding has proven useful to unveil the genetic diversity between closely related species, if not cryptic or semi-cryptic species of water mites, especially when obtained through the sequencing of the *cox1* gene. It is usually sufficient to perform molecular phylogenies within these species. For more distantly related species, e.g. belonging to different families, more conserved nuclear genes such as the small subunit of the ribosomal RNA gene are sometimes preferred^10,18,24^. What we would like to emphasize in our work is that complete mitogenomes can prove to be also useful. The amount of data retrieved from the concatenation of all protein-coding genes led to the obtention of a phylogenetic tree with optimal support at the nodes. The results obtained in this study combine geographically distant but taxonomically related species (*H. longiporus, H. taniguchii* and *H. turcicus*) into one clade, thereby establishing a sister group for the clade comprising the genus *Unionicola*, which belongs to the same superfamily (Hygrobatoidea) and contrasting the rest of the water mite species (*Sperchon clupeifer* and *Mideospis roztoczensis*). At the same time, all species of water mites constitute one group, a sister group of species belonging to Trombidia (genus Leptotrombium). This illustrates the great usefulness of complete mitogenomes for the recognition of relationship between geographically and taxonomically distant taxa.

Based on our results and subsequent comparisons with the works of other authors^22^, we could also notice some differences among the genus *Hygrobates* for what concerns the start and stop codons of their mitochondrial genes. In the near future, it would be interesting to sequence mitogenomes of other species closely related to *H. turcicus*, to see how much these features are conserved.

Finally, studies such as ours will be helpful in the near future for members of the community who work on biomonitoring based on metabarcoding or environmental DNA. We might cite the recent article from Blattner et al.^25^, which includes *Hygrobates norvegicus* among other bioindicator species. This study was based on the amplification of several mitochondrial genes. Sequencing complete mitogenomes of duly identified specimen of water mites will help documenting the databases for later uses in similar studies.

## Materials and Methods

### Biological material

The *H. turcicus* samples were collected from stones on the bottom of Bulgaria, Vidima River near Debnewo (42°56′41.4′′N, 24°48′44.6′′E, depth 0.4 m, water flow 0.71 m/s, temperature 10 oC, pH 8.53, oxygen 110%, conductivity 279 µS/cm, hardness 121 CaO mg/l), including 11 males, 27 females, 2 deutonymphs. Collected in 12.x.2019 by Zawal, Michoński & Bańkowska. One male and one female were dissected and slide-mounted for morphological identification.

### DNA extraction, sequencing, assembly and annotation

Water mites were collected by hand netting. Specimen were sorted out, initially identified and preserved in 96% ethanol, which is a method generally used in genetic research material^5^. Up to 50 specimens identified as *H. turcicus* were pooled together, and their DNA was extracted using the DNeasy Blood and Tissue Kit (Qiagen GmbH, Hilden, Germany) as described previously^23^. Exoskeletons were retrieved after DNA extraction and mounted in Hoyer’s medium*.* Sequencing was performed at the Beijing Genomics Institute in Shenzhen, China, on a DNBSEQ platform in accordance with the company's procedure. A total of ca. 40 million clean 150 bp paired-end reads were obtained and assembled using SPAdes 3.14.0^26^ using a k-mer of 125. The contig corresponding to the mitogenome was extracted, and the Consed^27^ package was used to verify its extremities. Annotations were done with the help of MITOS^28^ and manually curated.

### Comparison with other populations of *H. turcicus*

The *cox1* gene of *H. turcicus* was aligned with other 16 other sequences downloaded from GenBank and trimmed to a final size of 624 bp. The trimmed sequences were aligned on Clustal Omega online (ebi.ac.uk/Tools/msa/clustalo) to calculate the percentages of identities. We also computed the overall mean of genetic distances based on the Kimura 2-parameter model using MEGA 7.0. Standard error estimate(s) were obtained using bootstrap (1000 replicates).

### Multigene phylogeny

We aligned the 13 complete mitochondrial genomes with MAFFT version 7.510^29^, using *Riccardoella tokyoensis* and *Riccardoella reaumuri* as outgroup terminals. We conducted maximum likelihood (ML) analyses using RAxML-NG^30^ under three different strategies. (1) One of the IR regions was removed from all mitochondrial genomes to reduce overrepresentation of duplicated sequences before we ran RAxML-NG on the unpartitioned alignment under GTR + I + G substitution model as a single partition; (2) The same data was partitioned by gene, exon, intron and intergenic spacer regions, allowing separate base frequencies, α-shape parameters, and evolutionary rates to be estimated for each; (3) we inferred the best-fitting partitioning strategy with PartitionFinder2^31^ for the alignment. The best fitting nucleotide substitution models were inferred with jModelTest2^32^. Phylogenetic trees were visualized and edited with FigTree 1.4.4^33^. Support for the ML tree branches was calculated using the nonparametric bootstrap method with 1000 replicates.

## Data Availability

The complete mitogenome sequence of *Hygrobates turcicus* Pešić, Esen & Dabert, 2017 has been submitted to GenBank with the accession number OM336267. Data are available on Zenodo as the full sequence of the mitogenome in fasta format and annotations in tbl format with the following link: https://doi.org/10.5281/zenodo.6940457.
